# The Systemic and Pulmonary Immune Response to Staphylococcal Enterotoxins

**DOI:** 10.3390/toxins2071898

**Published:** 2010-07-21

**Authors:** Sanjeev Kumar, Antoine Ménoret, Soo-Mun Ngoi, Anthony T. Vella

**Affiliations:** Department of Immunology-MC3710, University of Connecticut Heath Center, 263 Farmington Avenue, Farmington, CT 06030, USA; Email: kumar@exchange.uchc.edu (S.K.); menoret@up.uchc.edu (A.M.); sngoi@student.uchc.edu (S.-M.N.)

**Keywords:** superantigen, T cells, staphylococcal enterotoxins

## Abstract

In response to environmental cues the human pathogen *Staphylococcus aureus* synthesizes and releases proteinaceous enterotoxins. These enterotoxins are natural etiologic entities of severe food poisoning, toxic shock syndrome, and acute diseases. Staphylococcal enterotoxins are currently listed as Category B Bioterrorism Agents by the Center for Disease Control and Prevention. They are associated with respiratory illnesses, and may contribute to exacerbation of pulmonary disease. This likely stems from the ability of Staphylococcal enterotoxins to elicit powerful episodes of T cell stimulation resulting in release of pro-inflammatory cytokines. Here, we discuss the role of the immune system and potential mechanisms of disease initiation and progression.

## 1. Introduction

Bacterial enterotoxins are proteinaceous toxins primarily ranging between 23–30 kDa that typically enter the body through mucosal sites. Unlike conventional antigens, enterotoxins can bypass antigen processing by binding to a specific groove outside the usual peptide-binding site of Major Histocompatibility class II (MHC II). These enterotoxins are often known as superantigens (SAgs) for their ability to bridge MHC II with the T cell receptor (TCR) unleashing robust T cell activation. In this review, we will examine the specificity of SAgs, their powerful effects on T cells, and their potential to induce strong pulmonary immune responses. The enterotoxins produced by *Staphylococcus aureus* will be emphasized since this pathogen is an emerging health concern in the living environment, hospitals, and as a co-infection.

## 2. Mechanism of Action on T Cells

Conventional antigen presentation requires protein processing and peptide binding to MHC I or MHC II followed by interactions with the combined interface of the TCRα and β chains. In contrast, SAg binds directly to the Vβ region of the TCR. Typically a particular SAg has specificity for more than one TCR Vβ chain and this is well documented for enterotoxins derived from *Staphylococcus aureus* (Table 1). Unlike MHC I and II restricted antigen presentation, both CD4 and CD8 T cells with specific TCR Vβ chains can be activated by SAgs. The oligoclonal nature of such stimulation activates a high proportion of T cells. Hence in human and mice, SAg elicits a powerful T cell response, characterized by robust proliferation and release of effector cytokines. Superantigen-stimulated T cells typically undergo 5–6 rounds of cell division [1] and activation status is clearly indicated by upregulation of surface CD69 [2]. Production of IFN-γ, TNF and other pro-inflammatory cytokines can be detected [1,2]. SEA-stimulated CD8 T cells exhibit cytotoxic killer phenotype that correlates with granzyme B and perforin expression [3]. Therefore, natural SAg responses are similar to artificial triggering by CD3 ligation.

**Table 1:** T cell receptor Vβ specificity of SE SAgs in mouse and human.

	**Mouse TCR Vβ**	**Human TCR Vβ**
**SEA**	1, 3, 10, 11, 17	1.1, 5.1, 5.2, 5.3, 6.3, 6.4, 6.9, 7.2, 7.3, 7.4, 7.9, 8, 9.1, 16, 18, 21.3, 22, 23.1
**SEB**	3, 7, 8.1, 8.2, 8.3, 17	1.1, 3.2, 6.4, 12, 13.2, 14, 15.1, 17, 20, 22
**SEC1**	3, 8.2, 8.3, 11, 17	3.2, 6.4, 6.9, 12, 15.1
**SEC2**	3, 8.2, 10, 17	12, 13.1, 13.2, 14, 15, 17, 20
**SEC3**	3, 7, 8.1, 8.2	5.1, 12
**SED**	3, 7, 8.1, 8.2, 8.3, 11, 17	1.1, 5.3, 6.9, 7.4, 8.1, 12.1
**SEE**	11, 15, 17	5.1, 6.1, 6.2, 6.3, 6.4, 6.7, 6.9, 8.1, 16, 18, 21.3
**SEG**		3, 12, 13.1, 13.2, 13.6, 14, 15
**SEH**		10
**SEI**		1.1, 5.1, 5.2, 5.3, 6b, 23.1
**SEJ**		
**SE*l*K**		5.1, 5.2, 5.3, 6.7, 21.3, 23
**SE*l*L**		5.1, 5.2, 5.3, 6.7, 7.9, 9, 16, 22, 23
**SE*l*M**		5.1, 5.2, 5.3, 6a, 6b, 7.1, 8, 9, 18, 21.3, 23
**SE*l*N**		5.1, 5.2, 5.3, 9, 20
**SE*l*O**		5.1, 7.1, 21.3
**SE*l*P**		5.1, 6, 8, 16, 18, 21.3
**SE*l*Q**		2.1, 5.1, 5.2, 6.7, 21.3
**SE*l*R**		3, 5.1, 8, 11, 12, 13.2, 14
**SE*l*U**		12, 13.2, 14
**SES**		9, 16
**SET**		
**TSST-1**	3, 15, 17	2.1

Summarized from: [18,124,125,126,127,128].

## 3. Binding of *Staphylococcus aureus* Enterotoxins to MHC II and the TCR

The primary peptide sequence of the SEs show significant variation [20], and the three dimensional structure of at least ten of them have been solved by crystallography: SEA [6], SEB [21,22], SEC2 [23], SEC3 [24], SED [10], SEG [25], SEH [26], SEI [27], SEK [28] and TSST-1 [29]. Although differences in SE structure are evident, the similarities are notable despite a lack of primary sequence homology. *Staphylococcal* enterotoxins are typically ellipsoid with two unequal domains including a B domain, which is the smallest and associated with carbohydrate binding. The characteristic SE disulfide bond is in the domain B and opposes the α-helical cap. The larger A domain contains both the amino and carboxyl ends and the interface between A and B contain α-helices which form a long groove on the back side of the molecule creating a shallow cavity at the top. 

The structural basis for SE immunological activity has been revealed by mutagenesis and confirmed by co-crystallization with TCR-β Chain [22,24,26,28,30]. The binding of SE to TCR depends on the shallow cavity at the top of the molecule. This cavity is thought to interact with three loops of the TCR Vβ, and the contact residues are from three distant regions of the primary sequence brought into proximity with each other in the folded protein. These amino acids are not highly conserved and may account for the SE specificity of Vβ. Crystallization of MHC II with SE reveal examples of MHC-peptide dependent binding [31,32,33], MHC-peptide independent binding [34], and finally both types of binding [35,36]. These mutational and functional analyses show structural dependencies concerning how SE activate T cells, which may provide new avenues for averting immune-based diseases mediated by SE. Lastly, recent comprehensive reviews detail the structural features of SAg and SAg like proteins including the properties of SAg binding to MHC and TCR [18,37].

## 4. How SE Affects Immune T Cell Tolerance

A hallmark of *in vivo* *S. aureus* enterotoxin responses is the manifestation peripheral T cell clonal expansion followed by deletion or programmed death of T cells responding to specific SAg [38,39]. This activation-induced cell death (AICD) response can be blocked when the host concomitantly responds to bacterial lipopolysaccharide [40], consistent with the recent finding that heightened IL-6 signaling can also inhibit SEB-induced T cell peripheral deletion [41]. While IL-6 signaling is known to induce naïve T cell survival [42], it was nevertheless shown that gp130 signaling (IL-6 receptor pathway) within SAg-specific T cells themselves does not block AICD [43]. They demonstrated that IL-6 signaling instead impeded Gr-1^+^ CD8 T cells from synthesizing IFN-γ, a cytokine central in SAg-based T cell tolerance [44]. Atsumi *et al.*, concluded that CD8 T cells synthesizing IFN-γ controlled CD4^+^ T cell AICD, but the effector molecule was unlikely to be IFN-γ since addition of IFN-γ did not increase cell death [43]. The death effector molecule remains an outstanding question in the *S. aureus* enterotoxin field, although using non-superantigen systems a combined role for both TNF receptor and CD95 for AICD of T cells has been documented *in vitro* [45], and *in vivo* [46].

Although *S. aureus* enterotoxins induce profound AICD there typically remains a subset of specific T cells that fail to delete [47,48] and their function has been the subject of many studies [49]. Perhaps their durability and anergic phenotype is an outcome of multiple TCR Vβ hits, but this effect is best observed after repeated injections of SEB. For example, three *in vivo* injections of SEB gives rise to CD4 CD25 T cells expressing Foxp3 protein, which can suppress via dendritic cells by the CD152 pathway [50]. A second study used a similar approach but discovered a novel CD25 negative CD4 T cell population expressing both Foxp3 and GRAIL [51]. Nevertheless, there is *in vivo* evidence for intrinsic anergy programming in the absence of Tregs after repeated SEB injection [52]. Thus, deletion and autonomous anergy are natural mechanisms that prevent hyper-responsiveness of T cells to *S. aureus* enterotoxins. An excellent clinical role for these pathways was recently revealed in a model of OVA-induced airway allergy [53]. The authors demonstrated that SEA pre-treatment in neonates enhanced OVA oral tolerance, which conferred protection against intranasal OVA challenge when the mice enter adulthood. The mechanism may involve an increase of CCR9-bearing Tregs but otherwise remains largely elusive. This is an illustrative example of how *S. aureus* (enterotoxin) can drive clinically beneficial tolerance. There are numerous examples of how SAgs are involved in disease progression [54], but a recent study demonstrated that orally-administered SEB mediated pathology in an adoptive transfer model of inflammatory bowel disease only when Tregs were absent [55]. Importantly, these results show that expansion of specific-TCR Vβ T cells in Chron’s patients may be a function of SAg exacerbating already established disease [56]. Therefore, *S. aureus* enterotoxins can induce Tregs for useful purposes, and conversely Tregs can also inhibit *S. aureus* enterotoxin mediated pathology.

## 5. The Mechanism of How SE Impacts Lung Immune Responses and Disease

The powerful immune response driven by SAgs discussed above poses other significant health risks to humans. Staphylococcal enterotoxins are currently listed on the CDC category B select agent list because of its potential to be used as a biological threat. Perhaps this is most evident by its propensity to rapidly mediate debilitating pulmonary responses after inhalation in people as observed in one known case of an accidental laboratory exposure of SEB [91]. The most recent developments concerning clinical issues, however, are results that link SE to asthma and other respiratory ailments [92]. It stands to reason that if SAgs drive such robust T cell responses in the periphery and intestinal mucosa, then their access to pulmonary sites is all the more serious. In general, this link is based on colonization of *S. aureus* in nasal polyps (NP) [93], which contain extensive expansion of skewed repertoires of TCR Vβ-possessing T cells [94]. In clinical studies this has been associated with severe upper and lower respiratory disease [95]. In fact, recent data show that some patients with chronic sinusitis/nasal polyposis produce IgE specific to *S. aureus* enterotoxins [96,97], and there is the same IgE association in some patients suffering from chronic obstructive pulmonary disease [98]. Secondly, the potential disease mechanism of *S. aureus* enterotoxin in nasal polyps may be the observed high level of IL-6 [99], which is known to contribute to Treg inhibition [100]. This is similar to human airway smooth cells which effectively present SE to T cells [101]. Importantly, human airway smooth cells synthesize IL-6 after CD40 stimulation [102], which is a key T cell based costimulatory pathway. Collectively, these human studies reveal the associated risks with exposure to SAg in pulmonary sites.

A very severe pulmonary illness difficult to study is the human medical condition status asthmaticus. Status asthmaticus is an acute and severe induction of asthma, and is thought to be triggered by a variety of lung irritants [103]. Ultimately, this condition is refractory to standard treatments such as steroids as recently reviewed [103]. Similar to patients experiencing a sudden asthmatic attack, which may occur in hours and be fatal [104], it is possible that disease onset is potentially linked to a role for SE as well as viral infection. A key observation in victims of fatal asthma is the appearance of CD8 T cells in the bronchial airways possessing cytotoxic ability as indicated by expression of perforin [105]. This is similar to a recent study examining peripheral blood responses in patients with mild to severe asthma during periods of exacerbation demonstrating that the patients with severe asthma possessed the highest levels of CD8 memory T cells with a Type 1 cytokine response as opposed to the expected Type 2 cytokine response [106]. New evidence shows that *S. aureus* which produce enterotoxins A-C can strongly stimulate IL-17 production by human memory T cells but not naïve [107], and this may contribute to toxic cytokine levels. Also, in a postmortem lung study of fatal asthma an increase of CD8 T cells containing cytotoxic abilities in bronchi were detected [105]. Consistent with CD8 T cell function and Type-1 cytokines a new report detected higher levels of nuclear NFκB in lung epithelial and parenchymal cells from patients that had died from fatal asthma compared to control lungs [108]. Lastly, the presence of a skewed TCR Vβ repertoire specific for SE in cells of bronchoalveolar lavage (BAL) from patients with poorly controlled asthma has been observed previously [109]. While these data are consistent with a potential link of SE or SAg playing a role in fatal asthma more proof will be needed and perhaps mouse models can play a role in helping clarify this issue. 

In mouse models of SEB intranasal (i.n.) administration, Herz *et al.*, demonstrated that SEB induces allergic airway disease in both BALB/c and C57BL/6 mice in a CD4^+^ T cell-dependent manner [110]. Importantly, repeated SEB i.n. exposure mediated classic allergic airway disease marked by increased inflammatory cells in BAL capable of producing pro-inflammatory cytokines, and enhanced airway responsiveness to methacholine challenge. This study provided an important link to human asthma and further demonstrated how SE can increase pulmonary inflammation. Further, a mouse expressing human HLA-DR3 extended these observations by showing that the higher dose of SEB dictates disease outcome from eosinophilic to neutrophilic infiltration [111]. The higher SEB i.n. dose (neutrophilic infiltration) caused systemic shock resulting in markedly increased levels of proinflammatory cytokines in serum and expansion of Vβ8 bearing T cells in spleen [112]. In addition, increased gene transcription of IL-5, IL-4, IFN-γ, IL-12 p40 in bronchi, elevated titers of OVA-specific and total IgE in serum, demonstrated that SEB exposure exacerbated or facilitated allergic inflammation in an airway disease mouse model perhaps through a dendritic cell-based process [113,114]. Therefore, SEB i.n. application in mouse provides a valuable research approach to model human pulmonary disease. 

Our recent work tested i.n. SEA exposure instead of SEB to examine other potential outcomes of pulmonary immune responses [115]. Histological evidence showed an impressive alveolitis marked by protein leakage as measured in BAL and increased levels of innate cells. Within days histopathology apparently resolved with decreases in protein and cells in BAL, but it is unclear if pathology such as collagen deposition would ensue as a result of this acute response. A striking accumulation of SEA-specific CD8 T cells in lung was evident, which was dependent upon CD4 T cells. Importantly, a concurrent accumulation of CD11c MHC II high cells dramatically increased in lung after i.n. SEA, which were shown to be largely derived from blood [116]. From a mechanistic perspective, it is clear that IFN-γ played a critical role in mediating alveolitis but not protein leakage. Thus, the i.n. SEA model show similarities to acquired respiratory distress syndrome or acute lung injury[117]. In a model of indirect acute lung injury CD4 T cells were specifically recruited to lung in an IL-16 dependent manner after hemorrhagic shock followed by septic challenge [118]. Importantly, these investigators found that Foxp3^+^ CD4 T regulatory cells played a key role in blocking neutrophil recruitment to lung via an IL-10 dependent process. These studies underscore the need for further investigation of systems that model human acute lung injury or acquired respiratory distress syndrome, which suggest a more prominent role for T cells in disease pathology.

## 6. Conclusions and Future Avenues of Investigation

Thus, the SE i.n. mouse models demonstrate how SAg stimulation of T cells affect airway, alveolar, and systemic sites of the body after inhalation exposure. Nevertheless, a number of important questions remain with perhaps the most obvious being how does a mitogenic T cell response cause such dramatic changes in the innate immune system as seen in the mouse models and human cases as discussed above. Certainly, it is reasonable to argue that a cytokine storm may play an important role, and there is a solid case for IL-2 being central to this idea. Recently, it was demonstrated that IL-2 deficient mice were resistant to SEB induced toxic shock unless the mice were reconstituted with IL-2 [119]. In addition, Chau *et al.*, demonstrated that toll-like receptor 2 (TLR2) ligands from the *Staphylococcal* cell wall inhibited SEB-based human T cell activation by reducing IL-2 production. This inhibitory effect resulted from a TLR2/peptidoglycan induction of IL-10, which also functioned in an *in vivo* mouse model [120]. This shows that IL-2 is central to a T cell-dependent cytokine shock response, but does not necessarily explain how this response conditions cells in the innate immune system. In particular, we found that after a SAg response, cells of the innate immune system possessed greater ability to respond to TLR ligands [121,122]. This conditioning was also evident through TLR pathways cross-talking to TLR non-responsive cells [123]. Therefore, in addition to cytokines it is possible that other factors are involved in accentuating the immune response beside IL-2.

We would like to suggest that another by-product of T cell stimulation such as the generation of endogenous danger-associated molecular patterns (DAMPS) may participate in amplifying a lung damaging response. This is perhaps a “T cell guided” innate response initiated by T cell killing and release of self proteins or lipids that are not normally detected in the steady state but when present are able to trigger inflammasome stimulation or TLR pathways in innate cells. This may produce a cytokine storm that most studies find and may in-turn further push innate cell activation. Second, the type and amount of DAMP generated may also be a biomarker of the magnitude of lung pathology and could perhaps be extrapolated to the seriousness of injury. An important point in this regard is that tissue fluid from the lung would likely have to be analyzed by techniques other than gene microarray since release of the DAMPs would most likely be a product of cell damage. While this idea is speculative it may be a pivot point to ascertain how a T cell mitogen can so potently condition the innate immune system. 
